# pMOSFETs Featuring ALD W Filling Metal Using SiH_4_ and B_2_H_6_ Precursors in 22 nm Node CMOS Technology

**DOI:** 10.1186/s11671-017-2080-2

**Published:** 2017-04-26

**Authors:** Guilei Wang, Jun Luo, Jinbiao Liu, Tao Yang, Yefeng Xu, Junfeng Li, Huaxiang Yin, Jiang Yan, Huilong Zhu, Chao Zhao, Tianchun Ye, Henry H. Radamson

**Affiliations:** 1 Key laboratory of Microelectronic Devices & Integrated Technology, Institute of Microelectronics, Chinese Academy of Sciences, Beijing, 100029 People’s Republic of China; 2 University of Chinese Academy of Sciences, Beijing, 100049 People’s Republic of China; 3 KTH Royal Institute of Technology, Brinellv. 8, 10044 Stockholm Sweden

**Keywords:** ALD W, High-*k* and metal gate (HKMG), Nano-beam diffraction (NBD), Threshold voltage (*V*_*t*_), Mobility

## Abstract

In this paper, pMOSFETs featuring atomic layer deposition (ALD) tungsten (W) using SiH_4_ and B_2_H_6_ precursors in 22 nm node CMOS technology were investigated. It is found that, in terms of threshold voltage, driving capability, carrier mobility, and the control of short-channel effects, the performance of devices featuring ALD W using SiH_4_ is superior to that of devices featuring ALD W using B_2_H_6_. This disparity in device performance results from different metal gate-induced strain from ALD W using SiH_4_ and B_2_H_6_ precursors, i.e. tensile stresses for SiH_4_ (~2.4 GPa) and for B_2_H_6_ (~0.9 GPa).

## Background

As continuous downscaling of complementary metal-oxide semiconductor (CMOS) into sub 20 nm nodes, strain engineering is utilized as an important technique to boost device performance [1]. There are a number of ways to exert strain to the channel, such as integrating SiGe or SiC as stressor material in source and drain region [2–6], stress memorization technology (SMT) [7], dual stress liners (DSL) [8], and metal gate stress technology (MGS). Among these techniques, MGS is attracting tremendous attention because of its easy integration with the state-of-the-art high-*k* and metal gate (HKMG)-last integration scheme and its effectiveness in inducing strain to the channel [9]. Initially, Intel utilized Al and TiN material as the filling metal in the gate region to induce compressive strain to enhance the performance of in 45 nm node n-MOSFET transistors [10]. However, as the aspect ratio of dummy gate trench became larger in 22 nm and beyond nodes, filling the trench without voids or seams by conventional Al metal confronted overwhelming challenge. Consequently, thanks to a good step coverage and conformity W metal using atomic layer deposition (ALD) emerges as a competitive candidate in filling the dummy gate trench [1, 11]. ALD W process was firstly developed by using precursors, Si_2_H_6_ and WF_6_ at 325 °C [12].

At this time, B-doped W metal layers using B_2_H_6_ and WF_6_ precursors have been systematically investigated by Kim et al. [13]. Later, more detailed studies about ALD W using SiH_4_ or B_2_H_6_ have been performed in terms of trench filling capability, threshold voltage vulnerability, and film adhesion during chemical mechanical polishing (CMP) [14–16]. However, ALD W as gate filling metal in real transistors and its impact on the channel stress is not systematically studied yet.

This work presents pMOSFETs of 25-nm gate length with HKMG-last and ALD W using SiH_4_ or B_2_H_6_ precursors as the gate filling metal. The effect of induced strain by metal gate on the performance of pMOSFETs featuring ALD W filling metal is also investigated. In this case, the impact of ALD W metal gate film stress modulation mechanism for device electrical performance could be discussed. This study can provide a foundation for ALD W film materials, which is very valuable for advanced transistor.

## Methods

**Fig. 1:**
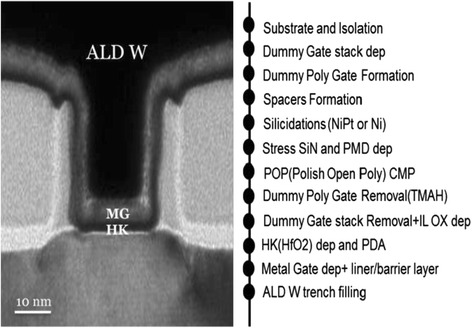
The fabrication process flow of pMOSFETs using HKMG-last integration scheme. Cross-sectional scanning electron microscopy images of fabricated pMOSFETs are also shown

The fabrication process flow of pMOSFETs is summarized in Fig. 1. The original material was 8-in. p-type (100) Si wafers. After the formation of N-well and shallow trench isolation (STI), dummy poly-Si gate of approximately 25-nm gate length was deposited and patterned by electron beam lithography (EBL). Followed by sequential spacer formation, Ni-Pt (5%) self-aligned silicidation, and the deposition of pre-metal dielectric, CMP to open the poly-Si dummy gate was performed. Upon removing the dummy gate by tetramethylammonium hydroxide (TMAH) and interfacial oxide layer by diluted HF, a 20-Å-thick HfO_2_ was deposited by ALD. Metal stack, i.e. ALD TiN/PVD Ti/CVD TiN, was then deposited as work function metals for pMOSFETs. Afterwards, 750-Å-thick ALD W films using SiH_4_ or B_2_H_6_ precursors were deposited to fill the gate trench. The ALD W films were deposited in Applied Centura iSPIRIT tungsten WxZ ALD chamber at 300 °C. The whole device fabrication was finished by metallization and forming gas annealing (FGA) at 425 °C.

At first, a few test samples were grown on blanket wafers containing two layers of TiN (10 nm)/SiO_2_ (300 nm)—followed by 75-nm ALD W film. The induced stress by ALD W films was evaluated by the difference in the radius of the wafer curvature. The difference in the radius of curvature before and after ALD W film deposition was carefully determined by laser reflection. X-ray diffraction (XRD) was performed to identify the phase of ALD W films. Cross-sectional transmission electron microscopy (TEM) images of fabricated pMOSFETs with ALD W as gate filling metal are also displayed in Fig. 1. The electrical characterization was carried out using a HP4156C precision semiconductor parameter analyser.

Nano-beam diffraction (NBD) technique in TEM was applied to provide advanced nano-scale information. These analyses were performed in combination with True Crystal Strain Analysis package program to find out the strain distribution along a vertical line starting from the channel region down to the areas deeper in the transistor body. The distributions of strain induced from W gate in the Si channel were studied using technology computer-aided design (TCAD) simulations.

## Results and Discussion

**Fig. 2:**
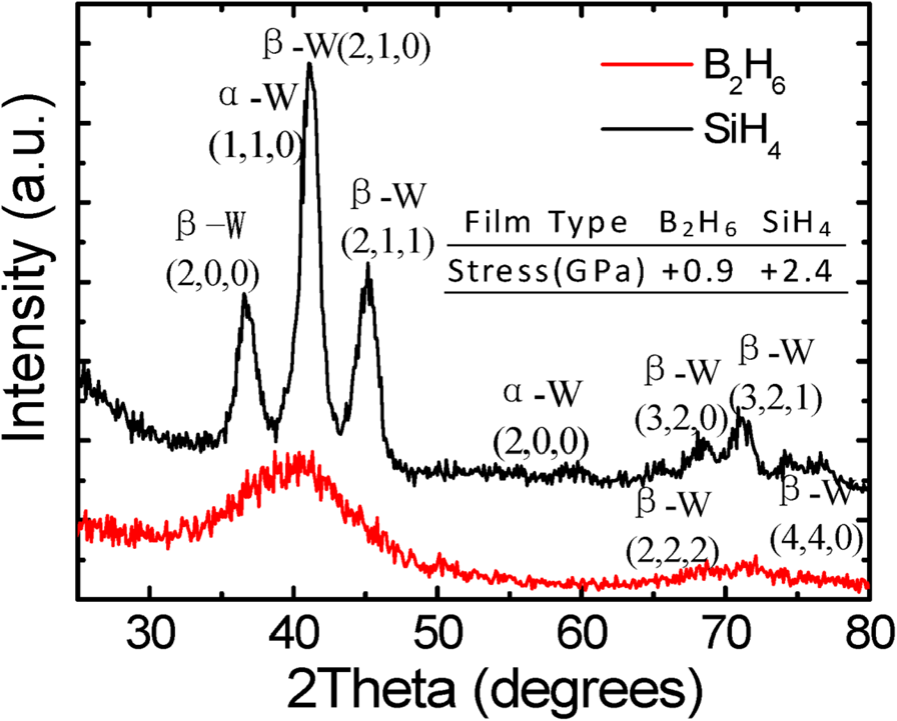
XRD spectra of ALD W using SiH_4_ and B_2_H_6_ and calculated stress data on blank substrate

In Fig. 2, the XRD spectra of ALD W using SiH_4_ and B_2_H_6_ and calculated stress data on blanket substrates are shown. It is seen that the ALD W using SiH_4_ has a higher tensile stress (~2.4 GPa) due to its polycrystalline phase whereas ALD W using B_2_H_6_ has a lower tensile stress (~0.9 GPa) due to its amorphous phase [17, 18]. Meanwhile, if these ALD W films with tensile stress are filled in the gate trench in a transistor structure, compressive strain along the channel direction will be induced. The ALD W filled at two sidewalls and at the bottom of gate trench tends to shrink and to “squeeze” two bottom corners, giving rise to compressive strain to the channel [19]. Consequently, enhanced hole mobility as well as improved electrical performance of as-fabricated pMOSFETs is realized, as will be elucidated later.

**Fig. 3:**
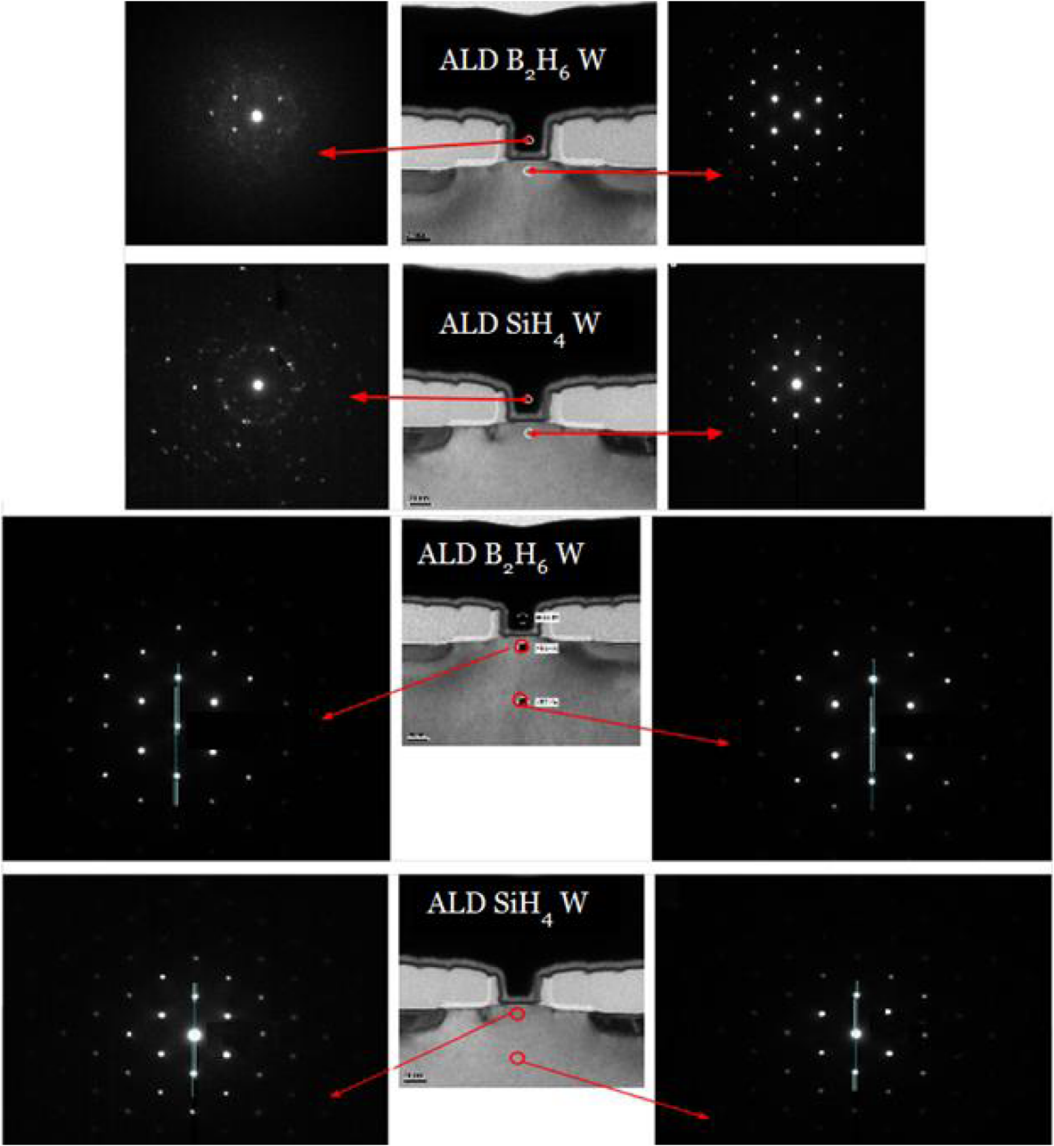
NBD images from metal gate, channel, and the reference regions of the transistors

The stress in the channel region was also measured directly on the transistor structures using NBD technique. Figure 3 shows the three sets of NBD images from device cross section including metal gate, channel, and reference regions of transistors where gate formed by ALD W using SiH_4_ and B_2_H_6_. The diffraction images from the metal gate materials show that Airy rings indicate polycrystalline material in agreement with XRD results. Meanwhile, ALD W using B_2_H_6_ has a pattern with weak intensity which is a sign of poor polycrystalline likely an amorphous phase.

In order to study the strain force from the W gate to Si channel, NBD analysis was performed and compared with a crystal part deep inside the transistor structure as a reference point. The idea behind NBD analysis is that the strain force causes a distortion of Si lattice constant or the change of interplanar distance of (220) planes. Therefore, a comparison between the measured and theoretically calculated data may reveal the stress amount. In this analysis, the software True Crystal program was applied to determine the lattice distortion. Later, the strain amount (*σ*) is converted into the stress (*ε*) by applying *ε* = *σ* / *E* where *E* is Young’s modulus. It is worth mentioning here that the source of strain is W gate but the strain in the Si channel is important. In this case, the applied *E* value for Si <100> direction (~200 GPa for a load amount of 15 mN) was used [20]. The estimated stress values were ~1 GPa for ALD W using SiH_4_ and ~0.5 GPa for ALD W using B_2_H_6_. The latter stress value is lower than the blanket samples measured by laser. A plausible reason may relate to strain relaxation during processing or sample preparation for TEM. But regardless to these reasons, the amount of stress in ALD W using SiH_4_ is almost double compared to ALD W using B_2_H_6_.

**Fig. 4:**
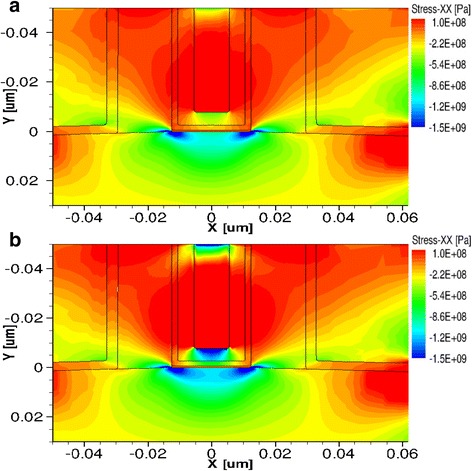
The TCAD simulation of strain distribution in the channel by **a** tensile ALD W using B_2_H_6_ and **b** tensile ALD W using SiH_4_ filled in the trench

TCAD simulation was performed to compare the strain effect by two ALD W metal electrodes filling in the trench, as shown in Fig. 4. The actual simulation parameters included the dimensions of pMOSFETs. The input parameters were 25 and 50 nm for the gate length and height, respectively. The other key process parameters were set according to the real device structure. The simulation results of stress profiles showed that the tensile ALD W using SiH_4_ has a higher strain in the channel region for high-*k* and metal gate-last pMOSFETs. It was seen that the channel strain profile is non-uniformly distributed in the channel region with compressive stress amount of ~0.7 and ~1.3 GPa for ALD W metal electrodes (ME) using B_2_H_6_ and SiH_4_, respectively.

**Fig. 5:**
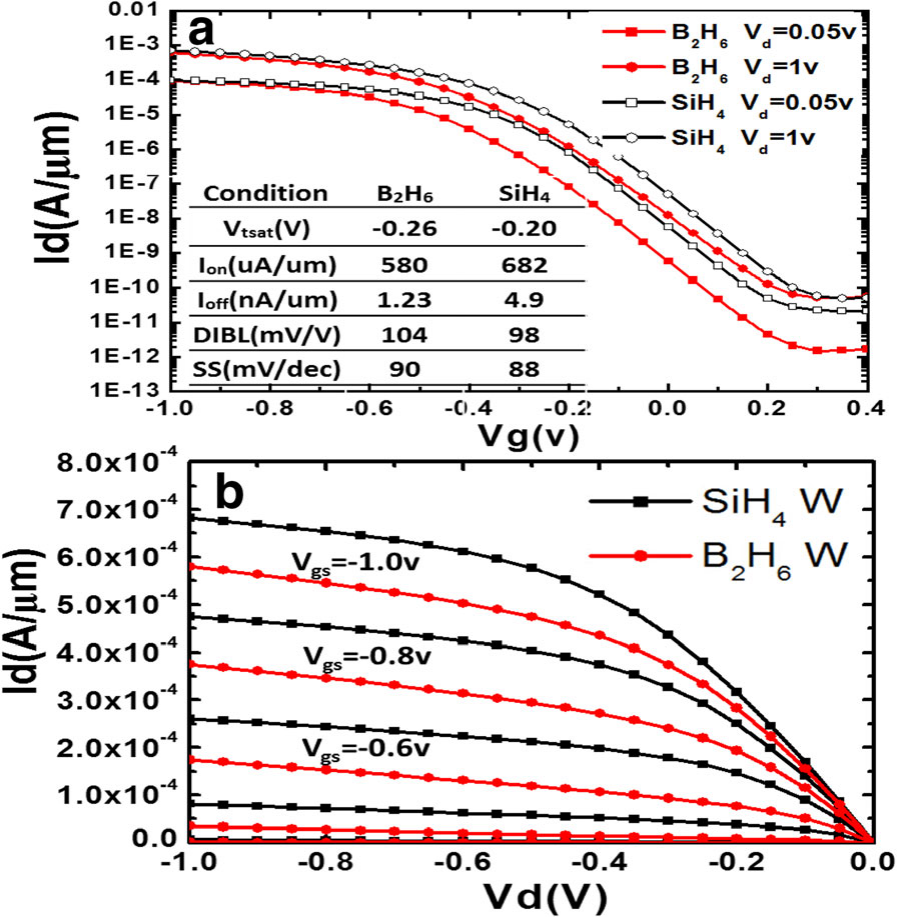
**a** *I*_d_-*V*_g_ and **b** *I*_d_-*V*_d_ characteristics of fabricated pMOSFETs with ALD W as gate filling metal. Basic device parameters are extracted and shown in the *inset* of **a**

For fabricated pMOSFETs with different ALD W as gate filling metal, the *I*_d_-*V*_g_ and *I*_d_-*V*_d_ characteristics are shown in Fig. 5. In the inset of Fig. 5a, basic device parameters are summarized. It is seen that the electrical performance of devices filled with different ALD W shows obvious deviations. Approximately 7% improvement of *I*_on_ can be accomplished for pMOSFETs filled with ALD W using SiH_4_ (703 μA/μm at *V*_ds_ = *V*_gs_ = −1.0 V), as compared to devices filled with ALD W using B_2_H_6_ (580 μA/μm at *V*_ds_ = *V*_gs_ = −1.0 V). The threshold voltage (*V*_*t*_), drain-induced barrier lowering (DIBL), and subthreshold swing (SS) for devices filled with ALD W using SiH_4_ are smaller, i.e. −0.20 V, 98 mV/V, and 88 mV/dec, respectively, than those for devices filled with ALD W using B_2_H_6_, i.e. −0.26 V, 104 mV/V, and 90 mV/dec, respectively. The superior driving capability and improved short-channel effect immunity as well as less negative *V*_*t*_ value for devices filled with ALD W using SiH_4_ than using B_2_H_6_ can be attributed to the strain effect. According to the deformation potential theory, the strain-induced bandgap narrowing, electron affinity, and density of states are the mainly reason for the *V*_*t*_ shift of MOSFETs [21]. The value of *V*_*t*_ shift depends on the amount of stress applied along the channel direction, especially for the channel compressed by a high stress [22]. It is worth noting that the shift of *V*_*t*_ to positive direction with large stress is consistent with previous work [23].

**Fig. 6:**
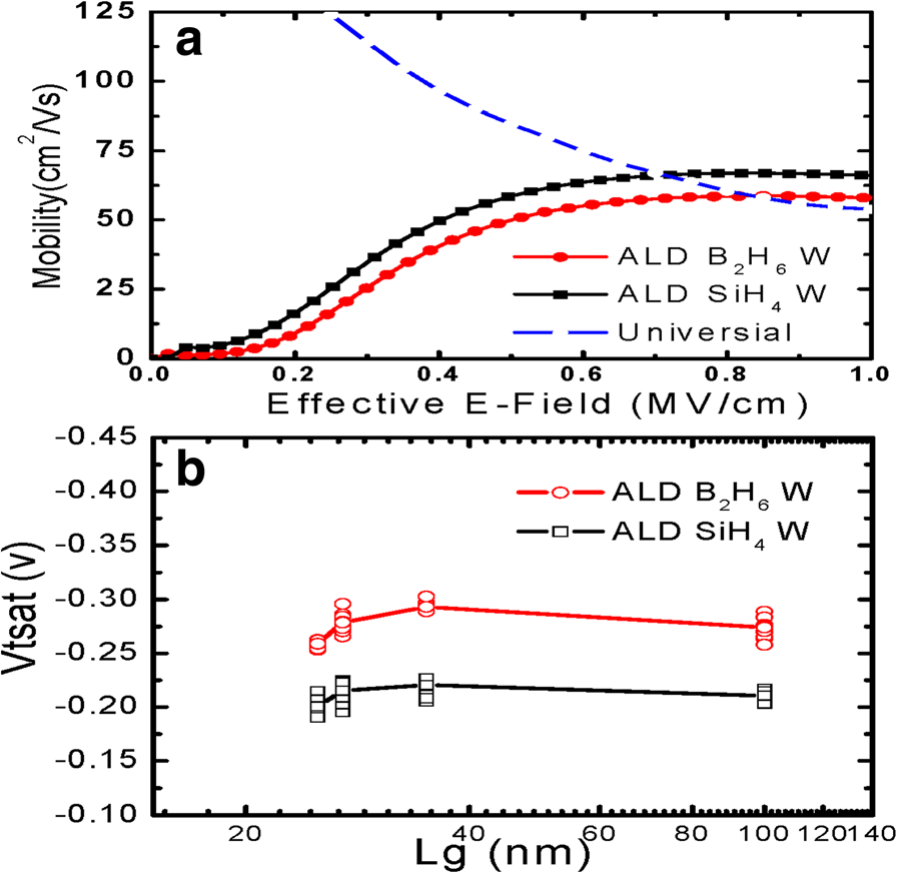
**a** Extracted carrier mobility and **b** *V*_*t*_ roll-off characteristics (*V*_d_ = −1 V) for pMOSFETs filled with ALD W using SiH_4_ and B_2_H_6_

In Fig. 6a, the mobility of pMOSFETs filled with different ALD W versus effective electrical field is shown. The figure shows that the mobility of devices filled with ALD W using SiH_4_ is 1.3 times larger than that using B_2_H_6_, which is also in good accordance with the larger stress in Fig. 2 as well as superior driving capability in Fig. 5. Compared to devices filled with ALD W using B_2_H_6_, the 30% improvement on mobility for devices filled with ALD W using SiH_4_, however, does not lead to equivalent improvement on *I*_on_. This can be described by the presence of the parasitic series resistance which counteracts the improvement on mobility for devices [24]. In Fig. 6b, the *V*_*t*_ roll-off characteristics of fabricated pMOSFETs as the shrinkage of gate length is displayed. For devices of all gate lengths filled with ALD W using SiH_4_, apart from lower *V*_*t*_ value, they show a better short-channel effect (SCE) immunity than devices filled with ALD W using B_2_H_6_. The *V*_*t*_ roll-off for the former is less significant than that for the latter. For the former devices with larger strain, less variation of bandgap reduction and stress-induced conduction band offset as the shrinkage of gate length could account for the less significant *V*_*t*_ roll-off [25].

## Conclusions

In summary, we investigated pMOSFETs featuring ALD W filling metal using SiH_4_ and B_2_H_6_ precursors. It was found that, compared to devices filled by ALD W using B_2_H_6_, devices filled by ALD W using SiH_4_ show higher drive capability and better control of short-channel effects. The on-current, DIBL, and SS for the latter are 703 μA/μm (*V*_ds_ = *V*_gs_ = −1.0 V), 98 mV/V, and 88 mV/dec, respectively. The superior device performance for devices filled by ALD W using SiH_4_ results from large compressive stress applied to the channel. Due to large stress as well as excellent trench filling capability of ALD W using SiH_4_, this technique, therefore, can be adopted extensively in the 22-nm and beyond node CMOS technology in the future.

## Abbreviations

ALD: Atomic layer deposition
DIBL: Drain-induced barrier lowering
DSL: Dual stress liners
EBL: Electron beam lithography
HKMG: High-*k* and metal gate
MGS: Metal gate stress technology
NBD: Nano-beam diffraction
pMOSFET: p-channel metal-oxide-semiconductor field-effect transistor
SCE: Short-channel effect
SMT: Stress memorization technology
STI: Shallow trench isolation
TEM: Transmission electron microscopy
TMAH: Tetramethylammonium hydroxide
XRD: X-ray diffraction
